# Comparison of tripterygium wilfordii multiglycosides and tacrolimus in the treatment of idiopathic membranous nephropathy: a prospective cohort study

**DOI:** 10.1186/s12882-015-0199-x

**Published:** 2015-12-04

**Authors:** Shanshan Liu, Xiayu Li, Heng Li, Qian Liang, Jun Chen, Jianghua Chen

**Affiliations:** Kidney Disease Center, The First Affiliated Hospital, College of Medicine, Zhejiang University, 79 Qingchun Road, Hangzhou, 310003 Zhejiang Province China

**Keywords:** Idiopathic membranous nephropathy, Tripterygium wilfordii Hook F, Tripterygium wilfordii multiglycosides, Tacrolimus

## Abstract

**Background:**

Idiopathic membranous nephropathy (IMN) is a major cause of nephrotic syndrome among adults. Considering the natural course of IMN, when to treat and with which immunosuppressive treatment need to be carefully considered in such patients. A combination of tripterygium wilfordii multiglycosides (TWG) and prednisone may be an effective option for treating patients with IMN.

**Methods:**

In this prospective cohort study, we enrolled patients with biopsy-proven IMN at our kidney centre. One cohort received TWG combined with prednisone, whereas another cohort received tacrolimus (TAC) combined with prednisone, for 36 weeks. The primary outcome was the remission rate, whereas the secondary outcomes included the time to remission, relapse rate, changes in serum albumin levels and daily urinary protein levels, estimated glomerular filtration rate, and adverse events.

**Results:**

A total of 53 patients with IMN met the criteria for enrolment, and all patients completed the therapy. At the end of the 36-week therapy, remission (either partial remission [PR] or complete remission [CR]) was observed in 20 patients (86.9 %) receiving TWG and in 27 patients (90.0 %) receiving TAC (*p* > 0.05), whereas CR was noted in 12 patients (52.2 %) receiving TWG and 14 patients (46.7 %) receiving TAC (*p* > 0.05). The probability of remission was similar for both the TWG and TAC groups (*p* > 0.05, by log-bank test). The mean time for achieving remission was 11.8 ± 12.5 weeks in the TWG group and 8.5 ± 9.1 weeks in the TAC group (*p* > 0.05).

**Conclusions:**

The combination of TWG and predisone is an effective and safe therapy for IMN.

## Background

Idiopathic membranous nephropathy (IMN) is one of the most common causes of nephrotic syndrome in adults. If no treatment is administered, IMN may lead to various outcomes. Although 30 % of patients with IMN experience spontaneously complete (CR) or partial remission (PR) [1], 30–40 % of patients develop end-stage renal disease (ESRD) within 5–15 years [2]. Considering the variable natural course of IMN, the time of treatment administration and the type of immunosuppression remain unclear.

According to the KDIGO (Kidney Disease: Improving Global Outcomes), a combination of corticosteroids and cytotoxic drugs (chlorambucil or oral cyclophosphamide) can induce remission of nephrotic syndrome in patients with IMN [3]. However, the possible side effects of this standard therapy, including myelosuppression, infection, and thrombosis, can lead refusal of the therapy by the patients [4, 5]. A recent placebo-controlled study suggested that tacrolimus (TAC) monotherapy was effective for IMN [6]. In a randomised control trial by *Min Chen et al.*, the remission rate was found to be higher and the decrease in urinary protein levels was found to be greater in IMN patients treated with TAC plus predisone as compared to those receiving cyclophosphamide (combined with predisone) [7]. In a previous study, we also observed that earlier initiation of therapy with TAC (combined with predisone) over 24 weeks was useful for ameliorating the severity of proteinuria in Chinese adults with IMN [8]. Although TAC can induce remission in most patients with IMN, the high relapse rates after treatment withdrawal, associated renal toxicity, and heavy cost burden are major concerns [9]. Hence, there is a need to explore alternative therapeutic strategies for IMN.

Tripterygium wilfordii Hook F (TwHF)—one of the most widely studied Chinese medicinal plants—is a member of the Celastraceae family of perennial vine-like plants. Tripterygium wilfordii multiglycosides is a preparation that is extracted and purified from the root xylem of TwHF [10], and is commercially available as tablets. Tripterygium wilfordii multiglycosides exerts both anti-inflammatory and immunosuppressive effects [11–13], and has been extensively used in China for the treatment of autoimmune diseases, such as rheumatoid arthritis [14], systemic lupus erythematosus (SLE) [15], and nephrotic syndrome [16]. Recent clinical studies indicated that TWG is a promising therapeutic option for patients with IMN [17].

In the present study, we evaluated the efficacy and safety of tripterygium wilfordii multiglycosides plus prednisone compared to those of TAC (combined with prednisone) in patients with IMN.

## Methods

This prospective cohort study was performed at a single centre, the Kidney Disease Center of the First Affiliated Hospital, College of Medicine, Zhejiang University (Hangzhou, P. R. China). All enrolled patients were admitted from January 2013 to December 2013. Before the treatments were initiated, we obtained written informed consent from the patients and approval from the ethics committee of our hospital (Medical Ethics Committee of the First Affiliated Hospital, College of Medicine, Zhejiang University). Patients were informed about the potential risks associated with all the drugs that they will receive.

### Study population

All the enrolled patients were older than 18 years and diagnosed with IMN by biopsy. We included patients who met the following criteria: (1) 24-hour urinary protein excretion of ≥3.5 g/d after 3–6 months observation of non-immunosuppressive therapy; (2) biopsy-proven membranous nephropathy; and (3) initial serum creatinine level of <133 μmol/L. We excluded patients if they met any of the following conditions: (1) previous treatment history with steroids or immunosuppressive medications; (2) patients who were not suitable for immunosuppressive treatment because of the presence of malignant tumour, gastrointestinal bleeding, human immunodeficiency virus or hepatitis B virus infection; (3) secondary membranous nephropathy, such as systemic lupus erythematosus, diabetes mellitus, or hepatitis B virus infection; and (4) serum creatinine (Scr) levels of ≥133 μmol/L.

### Indications for therapy

In this prospective cohort study, patients in the TWG group were treated with tripterygium wilfordii multiglycosides combined with oral prednisone, and patients in the TAC group received oral TAC combined with oral prednisone. Oral prednisone was initially prescribed at 0.5 mg/kg daily for 8 weeks, and the dose was reduced by 5 mg every two weeks to 20 mg; thereafter, the dose was maintained for 8 weeks and tapered over 16 weeks until complete withdrawal. In the TWG group, TWG was administered at 20 mg three times a day (totally 60 mg per day) during the initial 16 weeks; the dose was then reduced to 20 mg twice a day (totally 40 mg daily) and maintained for 8 weeks, followed by total withdrawal at the end of 36 weeks. In the TAC group, TAC was initially administered at 0.05 mg/kg daily, divided into 2 doses over 12-hour intervals. The dose was adjusted according to the trough blood level, with a target of 4–8 ng/ml, and was tapered at the beginning of the 12–24-week period according to the response; total withdrawal was ensured at the end of 36 weeks. With regard to patients with no response (NR) to the therapies or relapse during the therapy, those in the TWG group were recommended to quit the trial and undergo treatment with TAC and predisone, whereas those in the TAC group were recommended to quit the trial and undergo treatment with TWG and predisone. In our study, the multiglycosides preparation was made by Zhejiang DND Pharmaceutical Co., Ltd (Zhejiang, China)—each tablet contained 10 mg of the extract from the root xylem of Tripterygium wilfordii Hook F.

Follow-up was performed on a weekly basis for the first 4 weeks, after which follow-up was performed on a monthly basis. After the initiation of TAC treatment, we monitored the blood concentration of TAC in the patients each week and adjusted the dosage until stable levels of TAC were achieved. The serum levels of creatinine, glucose, albumin, alanine aminotransferase, and lipids, as well as complete blood counts and 24-hour urinary protein excretion were measured at each visit during the study period. The glomerular filtration rate (GFR) was estimated using the 4-variable Modification of Diet in Renal Disease (MDRD) study equation: GFR = 186 × serum creatinine^(−1.154)^ × age^(−0.203)^ × 1.212 (if African-American) × 0.742 (if female). Patients were also asked to report any adverse effects immediately or at each follow-up visit.

### Outcome variables and definitions

The primary outcome was the remission rate, including CR and PR. The secondary outcomes included the time to remission, relapse rate, changes in serum albumin levels, daily urinary protein levels, estimated glomerular filtration rate (eGFR), and adverse effects. CR was defined as a decrease in the daily urinary protein level to ≤0.3 g, whereas PR was defined as a reduction in the urinary protein level to 0.3–3.5 g/d along with a 50 % reduction from its peak values. Moreover, no response (NR) was defined as the persistence of nephrotic proteinuria after 12 weeks of treatment with TWG (60 mg per day) or TAC (with through blood level of 4–8 ng/mL). Time to remission was defined as the time from the initiation of therapy to the first day when CR or PR was observed. Relapses were defined as the observation of urinary protein levels in the nephritic range after a PR or CR, or an increase in the daily urinary protein levels by >50 % in patients who had previously shown a >50 % improvement in urinary protein levels without reaching a value of ≤0.3 g/day.

### Statistical analysis

Continuous variables are expressed as means ± standard deviation and categorical variables are presented as number/percentage. We compared the differences for normally distributed continuous variables between groups by using independent *t*-tests and non-normally distributed variables by using the Mann–Whitney test. Fisher’s exact test was used to compare the percentage of CR or PR in the 2 groups. The probability of remission or CR was analysed using Kaplan-Meier curves, and differences were estimated by using the log-rank test. All P values were 2-sided, and significance was set at a P value of 0.05. All the analyses were performed using SPSS version 19.0.

## Results

A total of 53 patients with IMN met the inclusion criteria, including 23 in the TWG group and 30 in the TAC group. The baseline characteristics in the 2 groups were similar (Table 1), and the patients were followed up until April 1, 2015. All patients completed the study and were included in subsequent analyses of efficacy and safety. The treatment protocol was switched in 8 patients (3 in the TWG group and 5 in the TAC group) because of relapse or NR (4 cases of NR, 4 cases of relapse) to the initial therapy.

**Table 1:** Baseline Characteristics of Patients Receiving Tripterygium Wilfordii Multiglycosides or Tacrolimus

	TAC group (n = 30)	TWG group (*n* = 23)	*p* value
Sex (female)	8(26.70 %)	11(47.80 %)	0.93
Age (years)	43.40 ± 16.10	48.82 ± 6.80	0.11
BMI (kg/m^2^)	23.79 ± 3.01	22.75 ± 3.16	0.28
Daily urinary protein (g)	6.32 ± 3.37	6.80 ± 2.25	0.54
Serum albumin (g/l)	23.51 ± 7.39	28.04 ± 9.86	0.06
Scr (μmol/l)	80.17 ± 30.24	66.04 ± 19.71	0.06
BUN (mmol/l)	5.96 ± 3.75	5.22 ± 2.73	0.43
UA (umol/l)	344.97 ± 136.04	340.65 ± 71.92	0.88
eGFR(MDRD) (ml/min)	83.70 ± 34.29	100.92 ± 28.27	0.06
Hb (g/l)	139.69 ± 17.19	133.74 ± 15.15	0.20
TG (mmol/l)	2.36 ± 1.82	2.57 ± 1.51	0.66
Tch (mmol/l)	8.38 ± 2.44	7.34 ± 2.38	0.13
LDL (mmol/l)	4.70 ± 0.61	4.88 ± 0.69	0.33
HDL (μmmol/l)	1.57 ± 0.48	1.56 ± 0.52	0.90
Blood glucose (mg/dl)	4.70 ± 0.61	4.88 ± 0.69	0.33
ALT (u/l)	24.40 ± 10.51	17.20 ± 6.18	0.15

Note: Data are expressed as mean ± standard deviation or number and (%). p values are calculated by analysis of variance, χ2 test, or independent *t*-test, as appropriate

*BMI* Body Mass Index; *Scr* serum creatinine; *BUN* blood urea nitrogen; *UA* Uric acid; *eGFR* estimated glomerular filtration rate; *Hb* haemoglobin; *TG* Triglyceride; *Tch* Total cholesterol; *LDL* Low density lipoprotein; *HDL* High density lipoprotein

### Response to therapy

**Fig 1:**
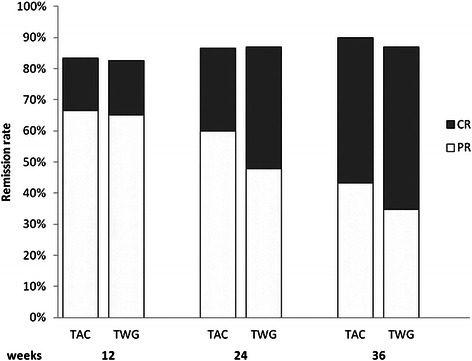
The percentage of remission (partial remission or complete remission) in patients receiving TWG or TAC during the 36 weeks of therapy, and the percentage of remission (either PR or CR) between the 2 groups were not significantly different (*p* > 0.05)

**Fig 2:**
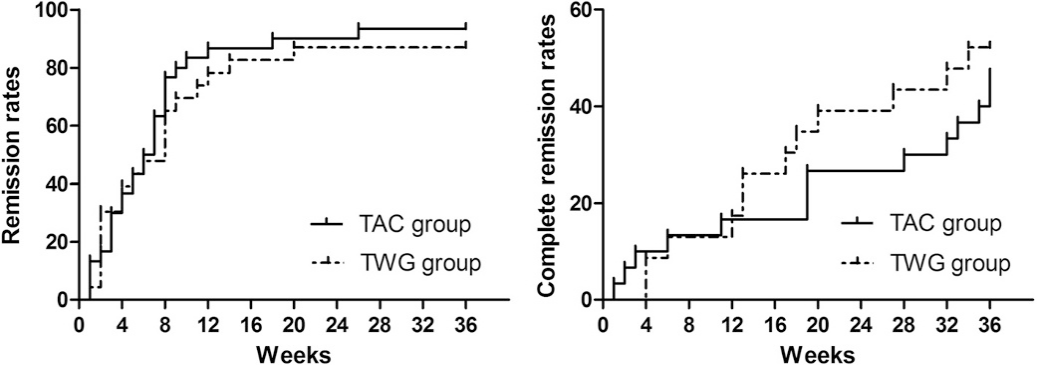
The probability of remission (either partial remission or complete remission) (log-rank test *p* = 0.42) and complete remission (log-rank test *p* = 0.57) in the TWG group and TAC group

CR and PR in patients in both groups during the 36-week therapy period are shown in Fig 1. During the 36-week therapy period, the percentages of remission (either PR or CR) in the TWG and TAC groups were 82.6 % and 83.3 % at 12 weeks (*p* > 0.05), 86.9 % and 86.6 % at 24 weeks (*p* > 0.05), and 86.9 % and 90.0 % at 36 weeks (*p* > 0.05), respectively. The percentages of CR in the TWG and TAC groups were 17.4 % and 16.7 % at 12 weeks (*p* > 0.05), 39.1 % and 26.7 % at 24 weeks (*p* > 0.05), and 52.2 % and. 46.7 % at 36 weeks (*p* > 0.05), respectively. The mean time to remission in the TWG group (11.8 ± 12.5 weeks; range, 1–36 weeks) was similar (*p* > 0.05) to that in the TAC group (8.5 ± 9.1 weeks; range, 1–36 weeks). The probability of remission (either CR or PR) and CR, estimated using the Kaplan-Meier method, showed no significant differences between the 2 groups (Fig 2). After 12 weeks of treatment, NR was recorded in 4 (17.4 %) patients in the TWG group and 4 (13.3 %) patients in the TAC group (*p* > 0.05). Among patients with NR, 4 (2 in TWG group and 2 in the TAC group) switched treatment to the corresponding protocol, but still did not achieve remission. The other 4 patients (2 in the TWG group and 2 in the TAC group) continued to receive the original therapy, including 3 (1 in the TWG group and 2 in the TAC group) who subsequently achieved PR and 1 (from the TWG group) who remained resistant to therapy.

Among the patients who at least experienced PR, 4 of 23 TWG patients (17.4 %) and 7 of 30 TAC patients (23.3 %) exhibited a relapse within the TWG or TAC treatment period (*p* > 0.05). The mean follow-up period after therapy was 10.6 ± 5.8 months (TWG group: 10.2 ± 6.3 months, range: 0.3–15 months; TAC group: 10.9 ± 5.5 months, range: 0.5–15 months). Moreover, relapse occurred in 4 (17.4 %) TWG patients and 6 (20 %) TAC patients (*p* > 0.05) during the follow-up period after cessation of treatment.

**Fig 3:**
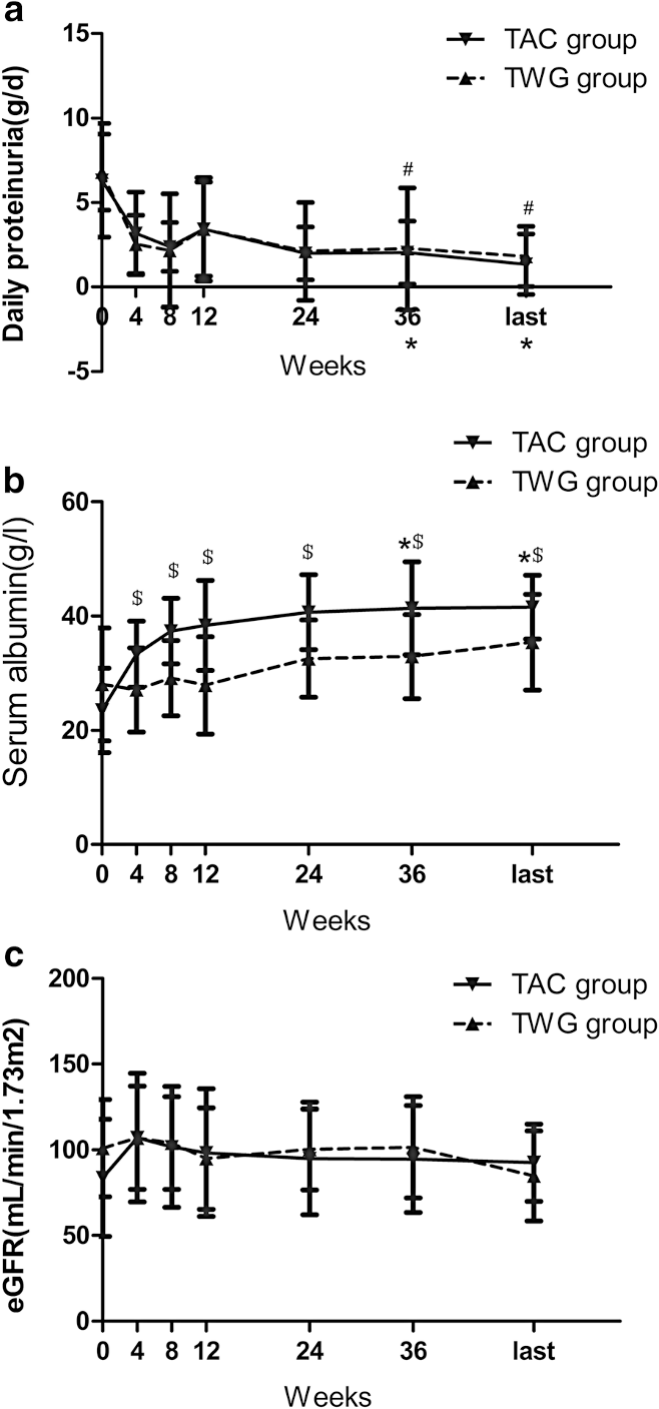
Time course of daily urinary protein levels, serum albumin levels, and eGFR levels in patients who received either TWG or TAC therapy. ^#^statistically a significant difference between the start and end of treatment in the TWG group (*p* < 0.05); *statistically a significant difference between the start and end of treatment in the TAC group (*p* < 0.05); ^$^statistically a significant difference between the 2 groups (*p* < 0.05). (**a**) Daily proteinuria of patients in the two group. (**b**) Serum albumin of patients in the two group. (**c**) eGFR of patients in the two group

Figure 3a and b show the daily urinary protein and serum albumin levels of patients before treatment (week 0), during therapy, and at the last follow-up. In the TWG group, 24-hour urinary protein excretion improved from 6.8 ± 2.3 g/day at baseline to 3.4 ± 3.1 g/day at week 12 (*p* < 0.05), 2.1 ± 2.9 g/day at week 24 (*p* < 0.05), and 2.3 ± 3.6 g/day at week 36 (*p* < 0.05), whereas serum albumin levels changed from 28.04 ± 9.86 g/l before treatment to 27.1 ± 8.5 g/l at week 12 (*p* > 0.05), 32.6 ± 6.8 g/l at week 24 (*p* > 0.05), and 32.9 ± 7.4 g/l at week 36 (*p* > 0.05). In the TAC group, the urinary protein levels decreased from 6.3 ± 3.4 g/day at baseline to 3.4 ± 2.8 g/day at week 12 (*p* < 0.05), 1.9 ± 1.6 g/day at week 24 (*p* < 0.05), and 2.1 ± 1.9 g/day at week 36 (*p* < 0.05), whereas serum albumin levels significantly increased from 23.5 ± 7.4 g/l at baseline to 38.4 ± 7.9 g/l at week 12, 40.7 ± 6.7 g/l at week 24, and 41.4 ± 8.1 g/l at week 36 (*p* < 0.05). There were no significant differences between the 2 groups in the change of daily proteinuria (*p* > 0.05), while the TAC group showed a significantly greater increase in serum albumin levels as compared to the TWG group (*p* < 0.05).

### Changes in renal function during follow-up

The average baseline eGFR was similar in the TWG group (100.92 ± 28.27 mL/min) and the TAC group (83.70 ± 34.29 mL/min) (Table 1). Figure 3c shows the eGFR of both groups before treatment (week 0), during therapy, and at the last follow-up (10.6 ± 5.8 months). The differences in the eGFR between the 2 groups during therapy and the follow-up periods were not significant (*p* > 0.05). At the final follow-up, the eGFR levels remained stable in all patients.

### Adverse effects

Among the 53 patients, 13 adverse events were recorded (TAC group, 7 events; TWG group, 6 events; Table 2). These side effects included gastrointestinal symptoms (3 in the TAC group and 2 in the TWG group), menstrual disorder (3 in the TWG group), anaemia (2 in the TAC group and 1 in the TWG group), and new-onset glucose intolerance (2 in the TAC group). All of the adverse effects were mild and well controlled.

**Table 2:** Adverse Effects in Patients Receiving Tripterygium Wilfordii Multiglycosides or Tacrolimus

	TAC group (n = 30)	TWG group (n = 23)
Leukopenia	0	0
Anaemia	2 (6.7 %)	1 (4.3 %)
New-onset glucose intolerance	2 (6.7 %)	0
New-onset hypertension	0	0
Liver injury	0	0
Infections	0	0
Bacterial pneumonia	0	0
Gastrointestinal symptoms	3 (10 %)	2 (8.7 %)
Menstrual disorder	0	3 (13.0 %)

## Discussion

IMN is an organ-specific autoimmune disease characterised by the subepithelial formation of immune deposits containing antigen, IgG, and complement components. The pathogenesis of IMN involves the formation of immune complexes and activation of the complement system; however, the specific mechanism remains unknown. Recently, Beck et al. reported that approximately 70 % of patients with IMN have circulating autoantibodies (mainly IgG4) directed against phospholipase A2 receptor 1 (PLA2R1), which is located on the surface of normal human podocytes, and induced proteinuria [18]. These findings facilitated progress in our understanding of the mechanism of IMN. Induction of remission in IMN patients can improve the quality of life of patients, and may also serve as a good predictor of the long-term outcome [19–21]. Various immunosuppressive regimens have been used in the treatment of IMN over the past 40 years. The decision of when to treat and with what regimen in a patient with IMN should consider the likelihood of a spontaneous remission as well as the potential adverse consequences of a exposure to immunosuppressants.

TAC is a drug of choice for IMN treatment. TAC does not only have an immunosuppressive effect on proteinuria, but may also exert an effect directly through cytoskeletal stabilisation in podocytes. However, the predominance effect of TAC is currently unclear. Compared with cyclosporine, TAC is a more potent inhibitor of antigen-driven T cell activation, cytokine production, and lymphocyte proliferation in vitro [22], and is associated with greater efficacy and less adverse effects in renal transplantation [23]. Previous studies have indicated that the TAC plus predisone regimen can induce a high remission rate in Chinese patients with IMN [7, 8], and is superior to the standard therapy involving alkylating agents [24]. Moreover, TAC is also effective in several resistant patients with IMN [25]. However, the relatively high cost of TAC and the various associated adverse events, such as drug-associated nephrotoxicity, have limited the use of TAC in patients with IMN. Hence, research on both valid and cost effective medications for the immunosuppressive treatment of IMN is ingoing, particularly in developing countries.

Tripterygium wilfordii multiglycosides is purified from TwHF, and has been widely used in China for the treatment of nephritis more than 30 years. Recently, a meta-analysis reported that TwHF has beneficial effects on the remission of idiopathic refractory nephrotic syndrome [25]. A randomised control trial conducted in China compared the effects of TWG monotherapy and combination therapy with TWG and predisone in patients with IMN. The results showed that TWG is a useful therapy to ameliorate proteinuria severity, and the effect of TWG combined with predisone is significantly greater than that of TWG monotherapy [17]. However, no study has compared the efficacy and safety of TWG (plus predisone) and TAC (plus predisone) in patients with IMN thus far. Among the 53 adults who received 36 weeks of therapy, 86.9 % of the patients in the TWG group and 90.0 % of patients in the TAC group achieved remission (either CR or PR); in particular, 12 of 23 patients (52.2 %) in the TWG group and 14 of 30 patients (46.7 %) in the TAC group experienced CR. The mean time to remission of the TWG protocol was similar to that of the TAC protocol. Most patients achieved remission during the first 12 weeks in both groups. We did not observe any significant difference in the relapses rates during or after treatment in the TWG or TAC group.

The multiglycosides preparation contains a variety of components, and the mechanisms by which it exerts its dramatic antiproteinuric effects on IMN is not fully explained. Evidence has suggested that triptolide is the most abundant constituent and is responsible for the immunosuppressive and anti-inflammatory effects [26–28]. Previous studies indicated that triptolide can inhibit interleukin (IL)-2 expression [29]; suppress the expressions of COX-2 and inducible nitric oxide synthase (iNOS) [30, 31]; and induce apoptosis in mitogen stimulated active T-cells, and arrest the growth of these T-cells [32]. It can also significantly inhibit the pro-inflammatory factor-induced up-regulation of class II MHC, B7-1, and B7-2 in the tubular epithelial cells [33], which are known to be involved in T-cell activation [34]. In addition to its immunosuppressive and anti-inflammatory activity, the therapeutic effect of triptolide may also be mediated through its direct activity on the restoration of podocyte injuries. In a recent report, the use of triptolide in animals with Heymann nephritis showed that triptolide could improve podocyte lesions and help restore the normal structure of the slit diaphragm [35]. Another study indicated that triptolide protects podocytes by inhibiting p-38 MAPK activation and fighting against the membrane attack complex (C5b-9) [36]. The mechanism of triptolide differs from that of prednisone [29], and is also distinct from that of calcineurin inhibitors (CNIs; cyclosporine and TAC) [37]. Triptolide was also more effective in preventing T cell proliferation and interferon-gamma production than FK506 [38]. Hence, triptolide can induce remission in patients who have no response to prednisone and CNIs.

Although the benefits of using TWG are apparent, this therapy has some side effects. The common adverse effects include upset stomach, liver injury, myelosuppression, reversible infertility in men, and amenorrhea in women [39, 40]. In the present study, menstrual disorders were detected only in the TWG group, but the medication was nevertheless well tolerated. The number of patients with new-onset glucose intolerance was greater in the TAC group. Moreover, it was observed that the serum albumin level in TWG group increased less than that in TAC group, and the underlying reason is unclear. An earlier report indicated that TAC can promote albumin synthesis in the liver [41], and we also speculate that TWG may affect the production of albumin in the liver. Hence, we carefully considered these effects when using TWG in patients with low serum albumin levels.

This study is limited by its nonrandomised format, and we suggest that larger prospective, randomised, multi-centre trials should be performed to evaluate the efficacy and safety of TWG in patients with IMN. Another limitation of this study is that it is unclear whether the findings can be applied to patients with IMN of other ethnic backgrounds, as the present study only included Chinese patients.

## Conclusion

Tripterygium wilfordii multiglycosides is highly effective for patients with IMN. The emergence of TWG therapy has actually reduced the heavy medical burden, because this medication is cost effective than other immunosuppressive remedies such as TAC, cyclosporine, and leflunomide, particularly for patients in developing countries. Therefore, TWG is a promising alternative therapy for patients with IMN.

## Abbreviations

TAC: tacrolimus
TWG: Tripterygium wilfordii multiglycosides
KDIGO: Kidney Disease, Improving Global Outcomes
BMI: Body Mass Index
Scr: serum creatinine
BUN: blood urea nitrogen
UA: Uric acid
eGFR: estimated glomerular filtration rate, calculated using 6-variable MDRD (Modification of Dietin Renal Disease) Study equation
Hb: haemoglobin
TG: Triglyceride
Tch: Total cholesterol
LDL: Low density lipoprotein
HDL: High density lipoprotein
